# A risk prediction model for heart failure hospitalization in type 2 diabetes mellitus

**DOI:** 10.1002/clc.23298

**Published:** 2019-12-14

**Authors:** Brent A. Williams, Daniela Geba, Jeanine M. Cordova, Sharash S. Shetty

**Affiliations:** ^1^ Department of Epidemiology and Health Services Research Geisinger Health System Danville Pennsylvania; ^2^ Boehringer Ingelheim Pharmaceuticals Ridgefield Connecticut

**Keywords:** diabetes, heart failure, risk prediction

## Abstract

**Background:**

Antidiabetic therapies have shown disparate effects on hospitalization for heart failure (HHF) in clinical trials. This study developed a prediction model for HHF in type 2 diabetes mellitus (T2DM) using real world data to identify patients at high risk for HHF.

**Hypothesis:**

Type 2 diabetics at high risk for HHF can be identified using information generated during usual clinical care.

**Methods:**

This electronic medical record‐ (EMR‐) based retrospective cohort study included patients with T2DM free of HF receiving healthcare through a single, large integrated healthcare system. The primary endpoint was HHF, defined as a hospital admission with HF as the primary diagnosis. Cox regression identified the strongest predictors of HHF from 80 candidate predictors derived from EMRs. *High risk* patients were defined according to the 90th percentile of estimated risk.

**Results:**

Among 54,452 T2DM patients followed on average 6.6 years, estimated HHF rates at 1, 3, and 5 years were 0.3%, 1.1%, and 2.0%. The final 9‐variable model included: age, coronary artery disease, blood urea nitrogen, atrial fibrillation, hemoglobin A1c, blood albumin, systolic blood pressure, chronic kidney disease, and smoking history (*c* = 0.782). High risk patients identified by the model had a >5% probability of HHF within 5 years.

**Conclusions:**

The proposed model for HHF among T2DM demonstrated strong predictive capacity and may help guide therapeutic decisions.

## 1 INTRODUCTION

Type 2 diabetes mellitus (T2DM) affects nearly 10% of the United States adult population, and the morbidity and mortality associated with T2DM are often attributable to cardiovascular (CV) disorders.^1^, ^2^, ^3^ T2DM is a strong predictor of new‐onset heart failure (HF) and HF‐related sequelae including hospitalization and death.^4^, ^5^ Beyond the well‐established association between T2DM and common HF antecedents such as atherosclerosis and myocardial infarction (MI), the metabolic aberration defining T2DM (elevated glucose) has been shown to have a direct and unique untoward effect on myocardial structure and function, with the term *diabetic cardiomyopathy* coined to describe the induced phenotype.^5^, ^6^, ^7^ Furthermore, in experimental settings, tight glucose control has been shown to improve both systolic and diastolic left ventricular function, implying a potentially direct beneficial impact of antidiabetic therapies on HF outcomes.^8^ However, randomized clinical trials have uncovered a wide range of effects (positive, negative, and neutral) of antidiabetic drug classes on HF outcomes, suggesting that off‐target, nonglucose‐related treatment effects may also be relevant among type 2 diabetics with or at risk for HF.^9^, ^10^, ^11^, ^12^, ^13^, ^14^, ^15^, ^16^

Given the strong association between T2DM, its therapies, and HF outcomes, it may be clinically valuable to identify type 2 diabetics at highest risk for HF outcomes to assist therapeutic decision making. Indeed, based on the aforementioned trial evidence, identifying patients at high risk for HF outcomes would have clear implications for antidiabetic therapy selection. Accordingly, the primary goal of the current study was to develop a prediction model for new hospitalization for heart failure (HHF) among type 2 diabetics initially free of HF. Secondary goals were to (a) identify and rank the strongest predictors of HHF in T2DM from a large, diverse set of candidate predictors, (b) develop a simplified scoring tool for facilitating application of the prediction model, and (c) propose a quantitative “high risk for HHF” probability threshold as a possible action point.

## 2 METHODS

This study incorporates the patient population and electronic medical record (EMR) data warehouse of a single integrated healthcare delivery system with a service area covering ~20,000 square‐miles in the northeast United States. Patients initially eligible for this study received primary care and other healthcare services through the study institution for at least 2 years between January 1, 2001, and November 10, 2015. Among patients meeting these criteria, type 2 diabetics were identified by any of the following: (1) observing the appropriate International Classification of Diseases—Ninth or Tenth Edition (ICD9/10) codes at two or more outpatient encounters at least 30 days apart but within one year (except in the context of a laboratory test order); (2) observing these ICD9/10 codes at one or more inpatient encounters; (3) when an oral antidiabetic drug (except metformin) was ordered or listed on a medication reconciliation; or (4) when metformin was ordered or listed on a medication reconciliation in the absence of a diagnostic code for prediabetes or polycystic ovary syndrome. Among patients meeting diagnostic criteria, an index date was defined as the date of the first office visit where T2DM diagnostic criteria were met at least two years following the first EMR‐documented encounter. Patients meeting the diagnostic criteria within two years of the first EMR‐documented encounter were considered to have pre‐existing T2DM at the index date, while those first meeting diagnostic criteria more than 2 years after the first EMR‐documented encounter were considered new diagnoses. Type 2 diabetics with documentation of HF prior to the index date were excluded. Follow‐up for the study outcome (HF hospitalization) continued through December 31, 2016. The study institution's IRB granted a waiver of patient consent due to the retrospective nature of the study.

A collection of 80 candidate predictors drawn from multiple EMR domains was considered for inclusion in the HHF prediction model (Table 1, excluding medications). Candidate predictors were determined through EMR documentation on or prior to the index date. Historical diagnoses, procedures performed, and CV‐related symptoms were determined through ICD9/10 and Current Procedural Terminology (CPT) codes documented at outpatient or inpatient encounters within the specified time window. For vital signs and laboratory tests, the value recorded in closest proximity to the index date was included. Laboratory tests considered in this study were hemoglobin A1c, basic metabolic panels, complete blood counts, liver function tests, and lipid profiles.

**Table 1:** Characteristics of type 2 diabetes mellitus patients at index date by occurrence of heart failure hospitalization during follow‐up

	All (n = 54 452)	HHF during F/U (n = 1884)	No HHF during F/U (n = 52 568)	*P*‐value
Incident T2DM (vs pre‐existing)	19 629, 36%	426, 23%	19 203, 37%	<.001
				
*Demographics and vital signs*				
Age (at index date), years	60 (50, 71)	69 (60, 76)	60 (50, 70)	<.001
Male	26 774, 49%	974, 52%	25 800, 49%	.03
White	52 536, 96%	1859, 99%	50 677, 96%	<.001
Smoking				
Current	9114, 17%	259, 14%	8855, 17%	<.001
Former	15 789, 29%	613, 33%	15 176, 29%	
Never	29 549, 54%	1012, 54%	28 537, 54%	
Body mass index, kg/m^2^	34 (29, 39)	33 (29, 39)	34 (29, 39)	.47
Systolic blood pressure, mm Hg	130 (120, 140)	137 (122, 150)	130 (120, 140)	<.001
Diastolic blood pressure, mm Hg	76 (70, 80)	72 (66, 80)	76 (70, 81)	<.001
Pulse pressure, mm Hg	54 (46, 64)	62 (50, 74)	54 (46, 64)	<.001
Mean arterial pressure, mm Hg	93 (87, 100)	94 (87, 102)	93 (87, 100)	<.001
Heart rate, bpm	76 (68, 84)	74 (66, 81)	76 (68, 84)	<.001
				
*Medical history*				
Anemia	8917, 16%	431, 23%	8486, 16%	<.001
Arrhythmia (non‐AF)	4240, 8%	233, 12%	4007, 8%	<.001
Atrial fibrillation	3042, 6%	284, 15%	2758, 5%	<.001
Cancer	7824, 14%	343, 18%	7481, 14%	<.001
Cardiomyopathy (non‐HF)	682, 1%	71, 4%	611, 1%	<.001
Cerebrovascular disease	4479, 8%	277, 15%	4202, 8%	<.001
Conduction disorder	1147, 2%	96, 5%	1051, 2%	<.001
CABG	2186, 4%	233, 12%	1953, 4%	<.001
Coronary artery disease	11 276, 21%	842, 45%	10 434, 20%	<.001
Dementia	823, 2%	27, 1%	796, 2%	.78
Depression	14 753, 27%	421, 22%	14 332, 27%	<.001
Family history of CVD	2756, 5%	46, 2%	2710, 5%	<.001
Gout	3544, 7%	179, 10%	3365, 6%	<.001
Hyperlipidemia	38 465, 71%	1341, 71%	37 124, 71%	.60
Hypertension	39 407, 72%	1597, 85%	37 810, 72%	<.001
Hyperthyroidism	745, 1%	24, 1%	721, 1%	.72
Hypothyroidism	9103, 17%	306, 16%	8797, 17%	.57
Implantable cardioverter defibrillator	126, <1%	21, 1%	105, <1%	<.001
Kidney disease (chronic)	5260, 10%	367, 19%	4893, 9%	<.001
Liver disease (chronic)	3120, 6%	62, 3%	3058, 6%	<.001
Lung disease (chronic)	10 748, 20%	430, 23%	10 318, 20%	<.001
Myocardial infarction	2938, 5%	217, 12%	2721, 5%	<.001
Pacemaker	643, 1%	69, 4%	574, 1%	<.001
PCI	2021, 4%	127, 7%	1894, 4%	<.001
Peripheral artery disease	3455, 6%	252, 13%	3203, 6%	<.001
Pulmonary embolism	727, 1%	35, 2%	692, 1%	.04
Sleep apnea	6042, 11%	183, 10%	5859, 11%	.05
Stroke/TIA	3209, 6%	165, 9%	3044, 6%	<.001
Thrombocytopenia	1016, 2%	59, 3%	957, 2%	<.001
Valve disease	2767, 5%	222, 12%	2545, 5%	<.001
Venous thromboembolism	1358, 2%	71, 4%	1287, 2%	<.001
				
*Cardiovascular symptoms*				
Angina/chest pain	13 304, 24%	512, 27%	12 792, 24%	.005
Dyspnea	7826, 14%	312, 17%	7514, 14%	.006
Edema	8202, 15%	372, 20%	7830, 15%	<.001
Fatigue	10 412, 19%	278, 15%	10 134, 19%	<.001
				
*Cardiovascular medications*				
Statin	26 320, 48%	960, 51%	25 360, 48%	.02
ACE inhibitor	24 535, 45%	1036, 55%	23 499, 45%	<.001
Diuretic	21 904, 40%	1050, 56%	20 854, 40%	<.001
Beta blocker	18 783, 34%	934, 50%	17 849, 34%	<.001
Antiplatelet (including aspirin)	17 059, 31%	606, 32%	16 453, 31%	.43
Calcium channel blocker	10 271, 19%	588, 31%	9683, 18%	<.001
Nitrate	6399, 12%	424, 23%	5975, 11%	<.001
Angiotensin receptor blocker	6993, 13%	335, 18%	6658, 13%	<.001
Warfarin	3275, 6%	243, 13%	3032, 6%	<.001
Fibrate	3554, 7%	130, 7%	3424, 7%	.50
Antiadrenergic antihypertensive	3097, 6%	175, 9%	2922, 6%	<.001
Intestinal chol absorption inhibitors	1600, 3%	51, 3%	1549, 3%	.54
Alpha‐beta blocker	1566, 3%	102, 5%	1464, 3%	<.001
Digoxin	1438, 3%	167, 9%	1271, 2%	<.001
Antiarrhythmic	1051, 2%	76, 4%	975, 2%	<.001
Nicotinic acid	956, 2%	38, 2%	918, 2%	.38
Bile acid sequestrants	912, 2%	43, 2%	869, 2%	.04
				
*Diabetes medications*				
Biguanides	26 722, 49%	769, 41%	25 953, 49%	<.001
Sulfonylureas	15 157, 28%	748, 40%	14 409, 27%	<.001
Insulin	9481, 17%	485, 26%	8996, 17%	<.001
Thiazolidinedione	5351, 10%	307, 16%	5044, 10%	<.001
DPP‐4 inhibitors	2184, 4%	55, 3%	2129, 4%	.01
Glitinides	1196, 2%	67, 4%	1129, 2%	<.001
				
*Laboratory tests*				
Hemoglobin A1c, %	6.9 (6.3, 8.0)	7.1 (6.4, 8.3)	6.9 (6.3, 8.0)	<.001
				
*Basic metabolic panel*				
Blood urea nitrogen, mg/dL	16 (13, 20)	19 (15, 25)	16 (13, 20)	<.001
Calcium, mg/dL	9.4 (9.1, 9.7)	9.4 (9.1, 9.7)	9.4 (9.1, 9.7)	<.001
Carbon dioxide, mEq/L	27 (25, 29)	27 (25, 29)	27 (25, 29)	.18
Chloride, mmol/L	102 (100, 104)	102 (99, 104)	102 (100, 104)	.16
Glomerular filtration rate, ml/min	60 (60, 60)	60 (60, 60)	60 (60, 60)	<.001
Glucose, mg/dL	138 (112, 176)	143 (113, 191)	138 (112, 175)	<.001
Potassium, mEq/L	4.3 (4.0, 4.6)	4.3 (4.0, 4.7)	4.3 (4.0, 4.6)	<.001
Sodium, mmol/L	139 (137, 141)	139 (137, 141)	139 (137, 141)	.003
				
*Liver function tests*				
Alanine aminotransferase, IU/L	25 (18, 38)	21 (16, 30)	26 (18, 38)	<.001
Albumin, g/dL	4.2 (4.0, 4.4)	4.0 (3.8, 4.2)	4.2 (4.0, 4.4)	<.001
Alkaline phosphatase, U/L	78 (64, 97)	79 (65, 100)	78 (64, 97)	.02
Aspartate aminotransferase, U/L	24 (19, 31)	23 (18, 29)	24 (19, 31)	<.001
Bilirubin, mg/dL	0.5 (0.3, 0.6)	0.5 (0.3, 0.6)	0.5 (0.3, 0.6)	.86
Protein, g/dL	7.1 (6.8, 7.5)	7.1 (6.7, 7.5)	7.1 (6.8, 7.5)	.006
				
*Complete blood count*				
Hematocrit, %	41.4 (38.5, 44.1)	40.1 (36.6, 43.2)	41.4 (38.6, 44.1)	<.001
Hemoglobin, g/dL	14.0 (12.9, 15.1)	13.6 (12.2, 14.7)	14.1 (13.0, 15.1)	<.001
Lymphocyte, % of total WBC	26 (20, 32)	23 (17, 30)	26 (20, 32)	<.001
MCHC, g/dL	34.0 (33.2, 34.6)	33.8 (33.0, 34.5)	34.0 (33.2, 34.6)	<.001
MCH, pg	30.3 (29.2, 31.5)	30.3 (29.0, 31.6)	30.3 (29.2, 31.5)	.38
Mean corpuscular volume, mcm^3^	89.4 (86.3, 92.4)	89.8 (86.7, 93.0)	89.4 (86.2, 92.4)	<.001
Mean platelet volume, fL	10.0 (8.8, 10.9)	10.2 (9.2, 11.0)	10.0 (8.8, 10.9)	<.001
Neutrophil, % of total WBC	63 (56, 70)	65 (58, 72)	63 (56, 69)	<.001
Platelet count, ×10^3^/mcL	240 (198, 289)	234 (187, 281)	241 (199, 289)	<.001
Red blood cell count, ×10^6^/mcL	4.6 (4.3, 5.0)	4.5 (4.1, 4.8)	4.6 (4.3, 5.0)	<.001
Red cell distribution width, %	13.4 (12.9, 14.1)	13.6 (13.0, 14.5)	13.4 (12.8, 14.1)	<.001
White blood cell count, ×10^3^/mcL	7.6 (6.3, 9.3)	7.8 (6.5, 9.5)	7.6 (6.3, 9.3)	.002
				
*Lipid panel*				
Cholesterol, mg/dL	185 (159, 213)	181 (155, 210)	185 (159, 213)	.001
HDL cholesterol, mg/dL	44 (37, 53)	44 (37, 52)	44 (37, 53)	.11
LDL cholesterol, mg/dL	102 (81, 127)	97 (77, 119)	102 (81, 127)	<.001
Triglyceride, mg/dL	166 (117, 239)	180 (124, 252)	166 (117, 238)	<.001
Total Chol: HDL	4.2 (3.4, 5.1)	4.1 (3.3, 5.1)	4.2 (3.4, 5.1)	.43
LDL: HDL	2.3 (1.7, 3.0)	2.2 (1.6, 2.8)	2.3 (1.7, 3.0)	<.001
Non‐HDL	138 (113, 167)	136 (111, 163)	138 (113, 167)	.008

*Note*: Continuous variables reported as median (interquartile range); categorical variables as n, %.

Abbreviations: ACE, angiotensin converting enzyme; AF, atrial fibrillation; CABG, coronary artery bypass graft; CVD, cardiovascular disease; DPP, dipeptidyl peptidase; F/U, follow‐up; HDL, high density lipoprotein; HF, heart failure; HHF, hospitalization for heart failure; LDL, low density lipoprotein; MCH, mean corpuscular hemoglobin; MCHC, mean corpuscular hemoglobin concentration; PCI, percutaneous coronary intervention; T2DM, type 2 diabetes mellitus; TIA, transient ischemic attack; WBC, white blood cell.

The study outcome was a new HHF, defined as an EMR‐documented, postindex date hospital admission with HF as the primary diagnosis in the absence of any prior documented HF diagnosis which constituted an exclusion criterion. A time‐to‐HHF variable was defined as the number of days from the index date until the first HHF or the last EMR‐documented encounter, with the latter defining censored observations. Cumulative incidence rates for HHF were estimated by the Kaplan‐Meier (KM) method for all study patients and repeated after stratifying by age and history of an HF diagnosis at the index date.

As a first step in the prediction model development process, T2DM patients meeting study inclusion criteria were randomly divided 1:1 into two independent data sets. Significant predictors of HHF were determined independently within each set using a similar variable selection process with the final proposed model combining the results of both sub‐models. In each set, a forward stepwise variable selection procedure was employed to identify the strongest independent predictors of time‐to‐HHF using Cox proportional hazards regression. A stringent *P*‐value threshold for variable inclusion/exclusion of .0001 was applied due to the large cohort and number of events. All continuous candidate predictors were categorized a priori into clinically relevant groups to facilitate model interpretation and development of an integer‐based risk score for HHF as described below. As missing data for vital signs and laboratory tests tend to be not missing at random (missing implies healthier),^17^ a conservative approach to missing data imputation was employed which involved taking a single random draw from the empirical distribution of nonmissing values for each continuous predictor. Drug therapies for CV disease or diabetes were not considered as candidate predictors as their effects may reflect confounding by indication.

Within each data subset, the variable selection process produced models with independent predictors each significant at a *P*‐value of .0001. For each model, the regression coefficients and 95% confidence intervals are reported, and the predictive strength of independent predictors ranked according to Wald chi‐square statistic magnitude from the multivariable Cox regression model. The discriminatory capacity of each model was quantified by the *c*‐statistic as appropriate for censored data.^18^ A final model was fit after restricting predictors to those meeting significance criteria in both models. An integer‐based risk score was created based on the regression coefficients from the final model. Integer scores were created for each variable in the final model by dividing each variable's regression coefficient by 0.243—the coefficient for the weakest predictor in the final model—and then rounding the quotient to the nearest integer. An integer risk score for HHF was created for each study patient by summing risk score components. The distribution of integer risk scores was divided at the approximate 50th, 75th, and 90th percentiles and labelled as low (<50th percentile), mid‐low (50th‐75th), mid‐high (75th‐90th), and high risk (>90th), respectively. That is, high risk was defined a priori, yet subjectively, as the 10% highest risk patients according to the prediction model. KM‐based estimated event rates among high risk patients were calculated so a high‐risk probability threshold could be proposed.

## 3 RESULTS

A total of 1,243,490 adult patients had at least one encounter at the study institution during the study interval, and 484,338 of these received primary care and other healthcare services through the study institution over a minimum 2‐year period. Among these, 59,180 (12%) met diagnostic criteria for T2DM. A history of HF at the index date was documented in 4728 (8%), thus the final study cohort consisted of 54,452. At the index date, 34,823 (64%) patients had pre‐existing T2DM and the remaining 19,629 (36%) were newly diagnosed. Median (IQR) age at the index date was 60 (50, 71) years, 49% were male, and 96% were of white race. CV risk factors were common, with 72% having hypertension, 71% hyperlipidemia, 21% coronary artery disease, and 5% prior MI (Table 1). Most CV risk factors and prior events were more frequent among those who experienced an HHF during follow‐up (Table 1). Median (IQR) hemoglobin A1c at the index date was 6.9% (6.3, 8.0), and blood glucose was 138 (112, 176) mg/dL.

**Figure 1:**
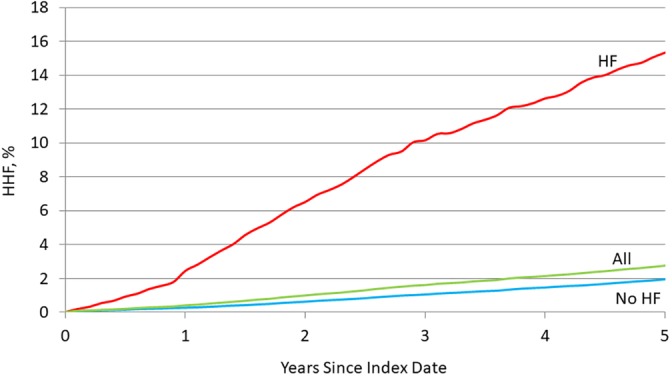
Cumulative incidence rates for heart failure hospitalization: overall and by history of heart failure at the index date

Type 2 diabetics with a prior HF diagnosis had a greater cumulative incidence of HHF than those without (Figure 1). Among type 2 diabetics without a prior HF diagnosis, a postindex date HHF occurred in 1884 (3.5%) study patients over 360,258 cumulative years of follow‐up (5.2 HHF per 1000 person‐years). Mean (SD) follow‐up among event‐free patients was 6.6 (4.3) years, and maximum follow‐up was 13.7 years. KM‐based cumulative estimated event rates at 6 months, 1, 3, and 5 years after the index date were 0.1%, 0.3%, 1.1%, and 2.0%, respectively (Figure 1). Event rates varied greatly across age groups (Figure S1).

The study cohort was randomly split into two equal subsets. In the first subset, among the original 80 candidate predictors, 14 were independently associated with HHF when a *P*‐value threshold of .0001 for model inclusion/exclusion was applied, with a *c*‐statistic of 0.785 (Table S1). In the second set, 15 predictors were independently associated with HHF with a *c*‐statistic of 0.804 (Table S1). Nine predictors were significant in both models, and after recombination of the data subsets the final prediction model with predictors in rank order of predictive strength included: (1) age, (2) coronary artery disease, (3) blood urea nitrogen, (4) atrial fibrillation, (5) hemoglobin A1c, (6) blood albumin, (7) systolic blood pressure, (8) chronic kidney disease, and (9) smoking history (Table 2). The final model had a *c*‐statistic of 0.782.

**Table 2:** Predictors of new heart failure hospitalization among type 2 diabetics—final model

Prediction model variable	Hazard ratio (95% CI)	Regression coefficient	Risk points
Age, years (vs < 40)			
40‐49	1.59 (1.10, 2.29)	0.465	2
50‐59	2.24 (1.60, 3.16)	0.808	3
60‐69	3.13 (2.23, 4.40)	1.142	5
70‐79	4.33 (3.08, 6.10)	1.467	6
≥ 80	8.05 (5.64, 11.50)	2.089	9
Coronary artery disease	2.20 (2.00, 2.42)	0.788	3
Blood urea nitrogen, mg/dL (vs 13‐16)			
17‐21	1.28 (1.12, 1.45)	0.243	1
22‐28	1.47 (1.28, 1.70)	0.387	2
≥29	2.59 (2.21, 3.03)	0.952	4
Atrial fibrillation	2.20 (1.93, 2.52)	0.790	3
Hemoglobin A1C, % (vs 6.0‐6.9)			
8.0‐8.9	1.63 (1.41, 1.89)	0.488	2
9.0‐9.9	1.79 (1.47, 2.17)	0.581	2
≥10.0	2.25 (1.91, 2.66)	0.813	3
Albumin, g/dL (vs ≥4.5)			
<3.5	2.40 (1.96, 2.95)	0.876	4
3.5‐3.9	1.85 (1.58, 2.16)	0.616	3
4.0‐4.4	1.32 (1.14, 1.52)	0.277	1
Systolic Blood Pressure, mm Hg (vs 110‐119)			
140‐149	1.50 (1.25, 1.79)	0.405	2
≥150	1.83 (1.54, 2.17)	0.602	2
Kidney disease	1.70 (1.50, 1.92)	0.529	2
Smoking Status (vs Never)			
Former	1.28 (1.15, 1.41)	0.243	1
Current	1.56 (1.36, 1.80)	0.447	2

**Figure 2:**
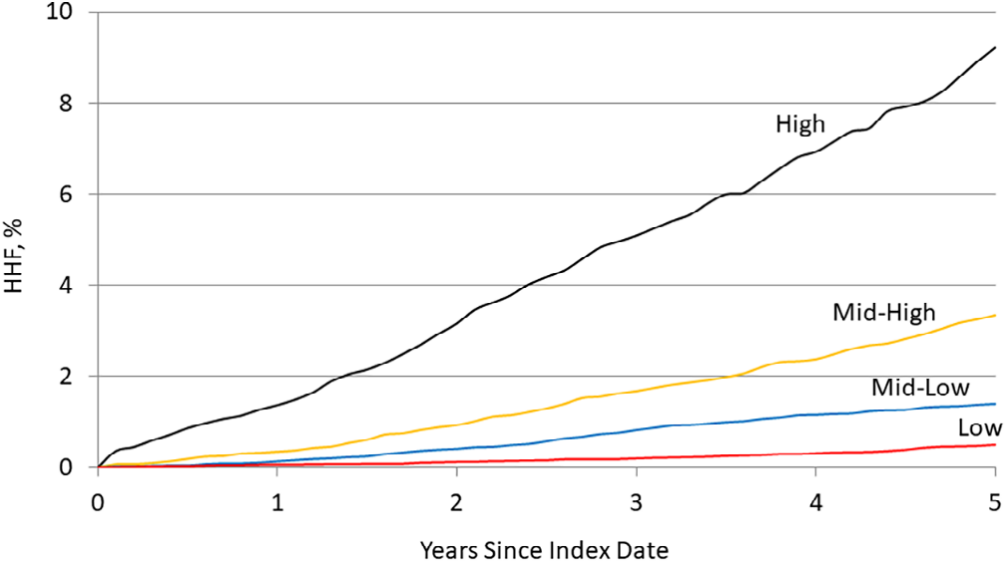
Cumulative incidence rates for heart failure hospitalization by risk score strata

Assignment of risk score points is shown in Table 2. The median (IQR) risk score for the final model was 9 (6, 12), and the maximum observed score was 29 (out of a possible maximum of 32) (Table 2). The number of risk points defining low, mid‐low, mid‐high, and high‐risk groups was ≤8, 9‐11, 12‐14, and ≥15, respectively (Supplemental Figure S2). The percent of patients at or above the respective risk scores is shown in Table 3. The observed 5‐year risk of HHF was above 5% for all risk scores 15 and above (high risk), and the estimated 1‐year risk was >0.5% (Table 3). Estimated event rates were widely divergent across risk strata (Figure 2).

**Table 3:** Kaplan‐Meier event rates by integer risk score

Score	n, %	% at or above	HHF event rates, % 1/3/5 years
≥22	302, 0.6%	0.6%	4.6/13.8/25.9
21	219, 0.4%	1.0%	3.8/9.1/18.4
20	296, 0.5%	1.5%	1.1/6.6/12.0
19	509, 0.9%	2.4%	2.6/8.6/15.0
18	690, 1.3%	3.7%	1.7/4.2/9.1
17	923, 1.7%	5.4%	1.6/6.8/11.4
16	1325, 2.4%	7.8%	0.6/3.5/6.2
15	1633, 3.0%	10.8%	0.6/2.6/5.5
14	2205, 4.0%	14.9%	0.5/2.6/5.0
13	2685, 4.9%	19.8%	0.4/1.6/3.2
12	3195, 5.9%	25.7%	0.2/1.2/2.4
11	3885, 7.1%	32.8%	0.3/1.3/1.8
10	4546, 8.4%	41.2%	0.1/0.8/1.5
9	4914, 9.0%	50.2%	0.1/0.5/1.0
8	5326, 9.8%	60.0%	0.1/0.4/1.0
7	5119, 9.4%	69.4%	<0.1/0.2/0.6
6	4964, 9.1%	78.5%	<0.1/0.2/0.5
5	4111, 7.6%	86.0%	<0.1/0.2/0.3
4	3060, 5.6%	91.6%	<0.1/<0.1/0.3
3	2551, 4.7%	96.3%	<0.1/<0.1/0.2
2	1004, 1.8%	98.2%	0/0/0.2
1	746, 1.4%	99.6%	0/0/0
0	244, 0.4%	100.0%	0/0/0

## 4 DISCUSSION

The current study developed an internally valid prediction model for new HF hospitalization among individuals with pre‐existing or new diagnoses of T2DM who had no prior documented history of HF. Prediction model variables were derived from the EMR of a single, large integrated healthcare system; thus, candidate predictors were necessarily limited to those gathered during usual clinical care. In the final 9‐predictor model, the strongest predictors of HHF in T2DM were (1) age, (2) coronary artery disease, (3) blood urea nitrogen, (4) atrial fibrillation, (5) hemoglobin A1c, (6) blood albumin, (7) systolic blood pressure, (8) chronic kidney disease, and (9) smoking history. The proposed model had a robust *c*‐statistic of 0.782. A quantitative threshold for labeling T2DM patients as at “high risk” for HHF was proposed as a >5% probability of HHF within 5 years—reflecting the 10% highest risk patients according to prediction model elements. Given the strong association between T2DM and HF, and the contrasting effects of different antidiabetic therapies on HF outcomes, the proposed prediction model may help guide antidiabetic drug selection in circumstances where HF warrants consideration.

Renewed interest in the diabetes‐HF link could be ascribed to the positive results from multiple clinical trials showing reductions in HHF with the sodium glucose cotransporter‐2 inhibitor class of diabetic drugs.^10^, ^16^ Notably, these beneficial effects were observed among patients both with and without established HF at randomization.^10^, ^16^ These findings contrast with previous trials showing adverse or neutral effects of other diabetic drug classes on HF outcomes.^9^, ^14^, ^19^ The importance of considering potential HF‐related effects of antidiabetic therapies is underscored by the high rates of HF risk factors among T2DM patients. Indeed, our study showed that 75% of T2DM patients had CAD, MI, and/or hypertension at the index date.

Though T2DM is strongly associated with a broad range of CV outcomes, and several prediction models exist for predicting CV risk in T2DM, multiple arguments can be put forth for creating a prediction model specifically for HHF.^20^, ^21^ First, an HHF is typically associated with an exacerbation of an underlying cardiac disorder, distressing symptoms, and portends an increased risk of short‐term mortality; thus, HHF is a serious health event warranting preventive focus.^14^, ^22^ Furthermore, T2DM is frequent among patients with an HHF, with up to 44% of hospital admissions related to HF reporting comorbid diabetes.^23^ HHF are costly to resolve, and readmissions are frequent, compounding the cost problem. Thus, aligning therapeutic management with future HHF risk allows prioritizing clinical resources toward objectively‐determined high risk patients where the HHF burden (and costs) are expected to be highest.^24^ Last, the detrimental (beneficial) effects of certain antidiabetic drugs have been most commonly ascribed to worsening (improving) fluid retention and congestion, the primary driver of HHF.^4^, ^25^ Accordingly, proper identification of at‐high‐risk‐for‐HHF patients may have implications for selecting appropriate therapy with a potentially significant impact on a frequent, distressing, and costly condition.

The *c*‐statistic in the current study was larger than typically observed with CV prediction models for T2DM at 0.782, possibly explained by a diabetic cohort both with and without known cardiac conditions at baseline, and consideration of an endpoint more amenable to prediction (HF, rather than MI or ischemic stroke).^20^, ^21^ Not surprisingly, multiple CV‐related phenotypes (CAD, atrial fibrillation), traditional CV risk factors (age, systolic blood pressure, smoking), and markers of kidney dysfunction (blood urea nitrogen, albumin) were independent predictors of HHF. Furthermore, the current study was unique in its ability to consider several blood biomarkers commonly measured in usual clinical practice among type 2 diabetics, and blood urea nitrogen, albumin, and hemoglobin A1c were among the final predictors. Notably, hemoglobin A1c was associated with HHF while blood glucose was not; this observation requires further investigation as hemoglobin A1c reflects longer‐term glycemic exposure while blood glucose reflects shorter‐term exposure.^26^, ^27^ Hemoglobin A1c has been found to be associated with HF in previous work.^28^, ^29^

The practical intent of risk prediction models is to identify high risk patients such that cost‐efficient provision of advanced management strategies (eg, a novel, efficacious, yet expensive pharmaceutical) can be directed toward those patients most likely to experience untoward events, thus minimizing the number needed to treat for benefit.^30^ Ultimately, a prediction model's quantitative output implicitly proposes an action (or not) by separating “high (enough) risk” (take action) from “not high (enough) risk” (do not take action) patients. The current study is the first to our knowledge to propose such an objective “high risk” threshold for HHF among type 2 diabetics: *a >5% probability of HHF within the next five years*—reflecting the 10% highest risk patients according to model predictors. Any *take action* threshold is preferably based on absolute risk estimates, thus stressing the importance of evaluating the calibration of the proposed model in new settings. Understanding that variation in what is judged “high risk” will exist, observed event rates for various risk scores (and percentiles) are provided in Table 3. Importantly, the proposed prediction model was developed using data generated from an EMR system which facilitates transporting the proposed model to other healthcare systems with a comparable data source and structure.

Some limitations of this study should be noted. The use of EMR data precludes consideration of certain candidate predictors either not generated during usual clinical care or not routinely available in structured form in our EMR (eg, duration of diabetes). Though the applied operational definitions for study variables were designed to minimize misclassification, measurement error, and missing data, these shortcomings are impossible to resolve completely, and the net effect is likely attenuation of hazard ratios. External validation of the proposed model should precede its application to ensure the model's discrimination and calibration are satisfactory in other settings. External validation is also vital due to the largely white patient population and limited geographical reach of the study institution.

In conclusion, the proposed 9‐predictor model for estimating HHF risk in T2DM showed strong predictive capacity. The proposed high‐risk threshold may serve as an action point for selection of antidiabetic therapeutics—a salient issue considering the opposing effects of different antidiabetic drug classes on HF outcomes.

## CONFLICT OF INTEREST

The authors declare no potential conflict of interests.

## DISCLOSURES

Boehringer Ingelheim Pharmaceuticals provided financial support for this study. J.M.C. and S.S.S. are employees of Boehringer Ingelheim Pharmaceuticals. B.A.W. receives research support from Biosense Webster, Roche, Gilead, Janssen, and Merck.

## Supporting information


**Figure S1:** Cumulative Incidence Rates for Heart Failure Hospitalization by Age at Index Date

**Figure S2:** Distribution of Risk Score Points and Proposed Risk Score Categories

**Table S1:** Prediction Models within Separate Randomly‐Split Analysis Sets
