# Supplementary figures and images for: A risk prediction model for heart failure hospitalization in type 2 diabetes mellitus
Source: Clin Cardiol. 2019 Dec 14;43(3):275–83. doi: 10.1002/clc.23298 (PMC7068070; doi:10.1002/clc.23298)

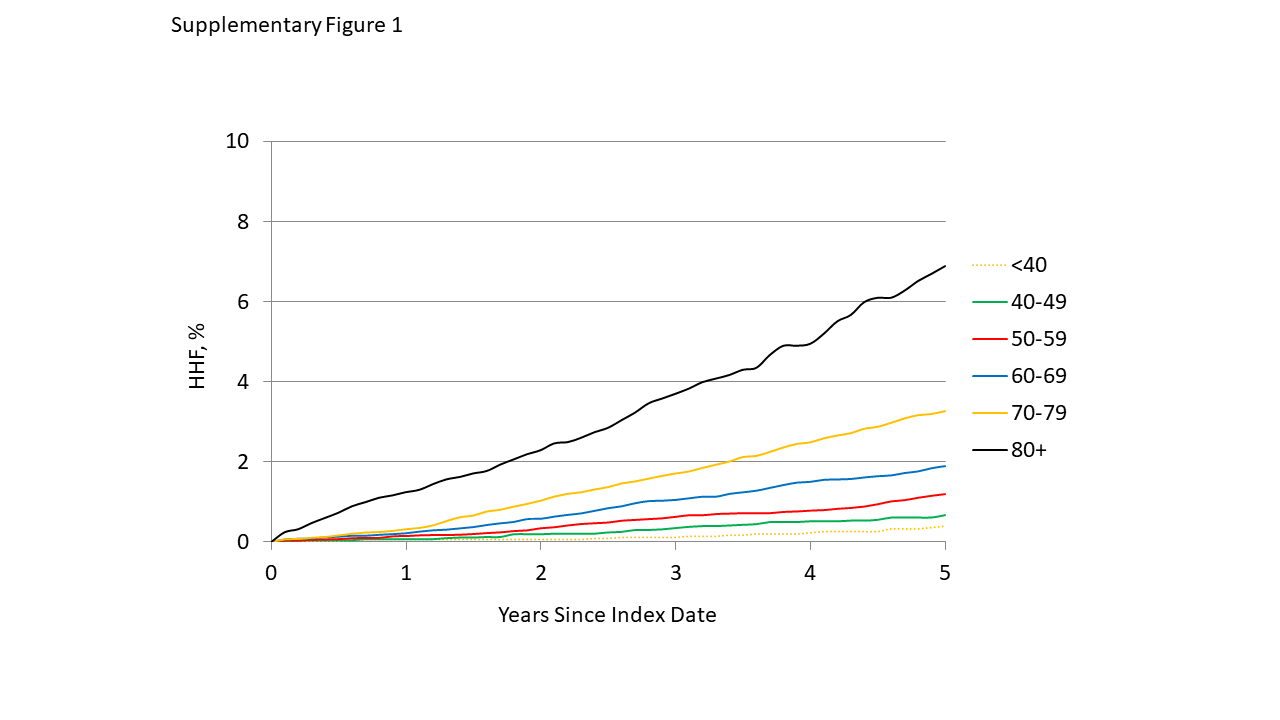

Supplement: Supplementary file 1 — Figure S1: Cumulative Incidence Rates for Heart Failure Hospitalization by Age at Index Date [file CLC-43-275-s001.tif]

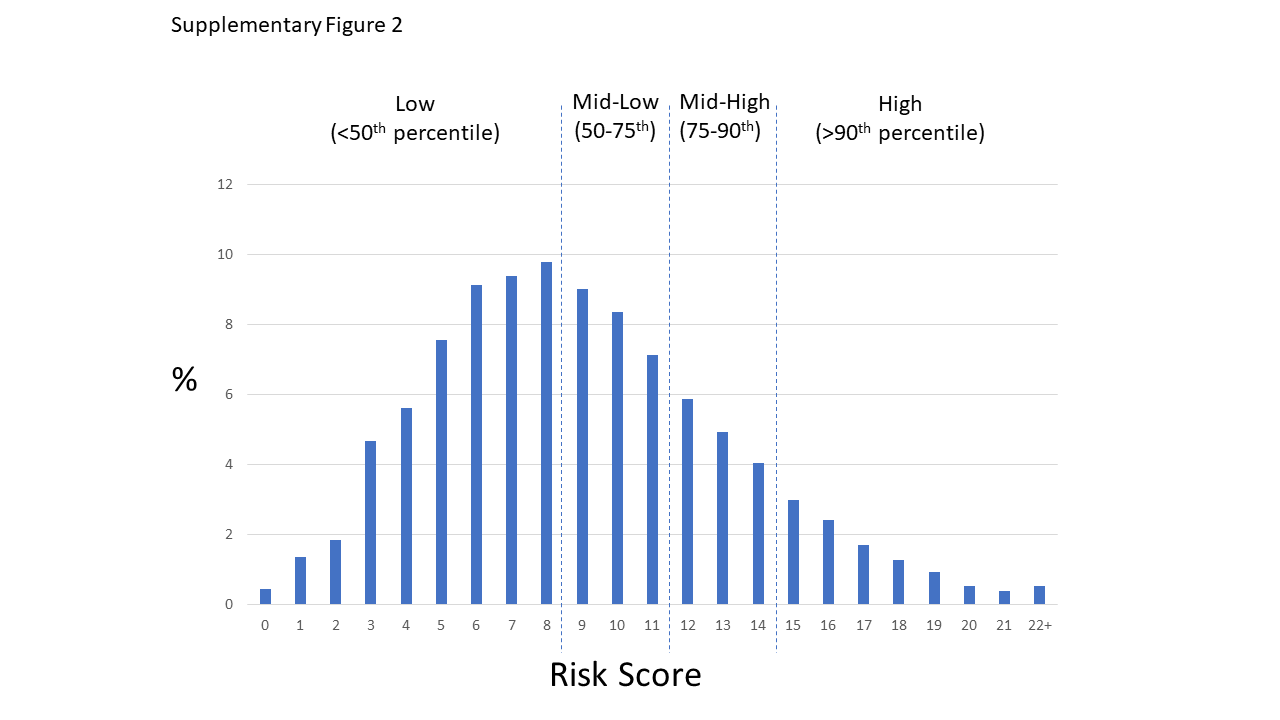

Supplement: Supplementary file 2 — Figure S2: Distribution of Risk Score Points and Proposed Risk Score Categories [file CLC-43-275-s002.tif]
