# Supplementary material for: A risk prediction model for heart failure hospitalization in type 2 diabetes mellitus
Source: Clin Cardiol. 2019 Dec 14;43(3):275–83. doi: 10.1002/clc.23298 (PMC7068070; doi:10.1002/clc.23298)
Supplement: Supplementary file 3 — Table S1: Prediction Models within Separate Randomly‐Split Analysis Sets [file CLC-43-275-s003.docx]

Supplementary Table: Prediction Models within Separate Randomly-Split Analysis Sets

|  | Set #1  HR (95% CI) | Set #2  HR (95% CI) | Rank  Set #1 | Rank  Set #2 |
| --- | --- | --- | --- | --- |
| Age (vs. <40)  40-49  50-59  60-69  70-79  ≥80 | 1.61 (0.96, 2.71)  2.57 (1.59, 4.17)  3.45 (2.14, 5.58)  4.41 (2.71, 7.15)  8.25 (4.98, 13.66) | 1.63 (0.98, 2.74)  2.27 (1.40, 3.70)  3.46 (2.13, 5.60)  5.42 (3.32, 8.82)  9.62 (5.77, 16.06) | 1 | 1 |
| Coronary Artery Disease | 1.94 (1.68, 2.23) | 2.34 (2.04, 2.69) | 2 | 2 |
| Blood Urea Nitrogen, mg/dl (vs. 13-16)  <11  11 – 12  17 – 21  22 – 28  ≥29 | 1.04 (0.75, 1.45)  1.20 (0.92, 1.58)  1.43 (1.20, 1.72)  1.47 (1.19, 1.81)  2.68 (2.14, 3.36) | 1.12 (0.83, 1.52)  0.97 (0.72, 1.29)  1.13 (0.94, 1.36)  1.43 (1.17, 1.75)  2.18 (1.74, 2.74) | 3 | 3 |
| Hemoglobin A1C, % (6.0-6.9)  <6.0  7.0-7.9  8.0-8.9  9.0-9.9  ≥10.0 | 1.03 (0.82, 1.28)  1.32 (1.12, 1.56)  1.69 (1.37, 2.09)  2.07 (1.58, 2.71)  2.08 (1.63, 2.65) | 1.05 (0.84, 1.30)  1.01 (0.85, 1.21)  1.42 (1.14, 1.76)  1.30 (0.97, 1.76)  1.91 (1.47, 2.49) | 4 | 8 |
| Atrial Fibrillation | 2.06 (1.70, 2.48) | 1.94 (1.60, 2.36) | 5 | 4 |
| Albumin, g/dl (vs. ≥4.5)  <3.5  3.5 – 3.9  4.0 – 4.4 | 2.21 (1.65, 2.95)  1.85 (1.49, 2.30)  1.26 (1.03, 1.54) | 1.99 (1.47, 2.70)  1.58 (1.26, 1.98)  1.30 (1.06, 1.59) | 6 | 13 |
| Systolic Blood Pressure, mmHg (vs. 110-119)  <110  120-129  130-139  140-149  ≥150 | 1.20 (0.85, 1.71)  1.31 (1.02, 1.69)  1.33 (1.03, 1.72)  1.59 (1.22, 2.06)  1.97 (1.53, 2.54) | 1.13 (0.80, 1.59)  1.06 (0.83, 1.36)  1.13 (0.88, 1.45)  1.44 (1.12, 1.84)  1.76 (1.38, 2.23) | 7 | 6 |
| Chronic Kidney Disease | 1.54 (1.29, 1.84) | 1.77 (1.49, 2.11) | 8 | 5 |
| Smoking Status (vs. Never)  Former  Current | 1.34 (1.16, 1.55)  1.44 (1.17, 1.76) | 1.21 (1.04, 1.40)  1.84 (1.51, 2.25) | 9 | 7 |
| Anemia | 1.47 (1.25, 1.72) |  | 10 | - |
| Implantable cardioverter defibrillator | 3.66 (2.02, 6.61) |  | 11 | - |
| Cardiomyopathy (non HF) | 2.03 (1.46, 2.84) |  | 12 | - |
| Dyspnea | 1.43 (1.21, 1.70) |  | 13 | - |
| Hyperlipidemia (No vs. Yes) | 1.36 (1.18, 1.58) |  | 14 | - |
| Glucose, mg/dl (vs. 110-139)  <110  140 - 169  170 - 199  200 - 229  ≥230 |  | 1.55 (1.28, 1.88)  1.28 (1.04, 1.57)  1.62 (1.28, 2.05)  1.61 (1.21, 2.14)  1.60 (1.24, 2.06) | - | 9 |
| Pacemaker |  | 2.41 (1.75, 3.33) | - | 10 |
| Body Mass Index, kg/m^2^ (vs. 25 - <30)  <25  30-<35  35-<40  40-<45  ≥45 |  | 1.04 (0.79, 1.38)  1.18 (0.98, 1.42)  1.35 (1.10, 1.66)  1.50 (1.18, 1.91)  1.76 (1.38, 2.25) | - | 11 |
| Chloride, mmol/l (vs. ≥ 104)  <100  100-101  102-103 |  | 1.56 (1.30, 1.87)  1.22 (1.01, 1.46)  1.04 (0.86, 1.25) | - | 12 |
| Red blood cell count, x10^6^/mcl (vs. 4.5-4.9)  <4.0  4.0-4.4  ≥5.0 |  | 1.55 (1.27, 1.90)  1.35 (1.14, 1.60)  1.38 (1.14, 1.66) | - | 14 |
| Red cell distribution width, % (vs. <13.0)  13.0-13.9  14.0-14.9  ≥15.0 |  | 1.13 (0.95, 1.34)  1.42 (1.17, 1.73)  1.57 (1.27, 1.96) | - | 15 |
